# Seasonal variance of 25-(OH) vitamin D in the general population of Estonia, a Northern European country

**DOI:** 10.1186/1471-2458-9-22

**Published:** 2009-01-19

**Authors:** Mart Kull, Riina Kallikorm, Anu Tamm, Margus Lember

**Affiliations:** 1 Department of Internal Medicine, University of Tartu, Puusepa Str. 6, Tartu, Estonia; 2 Tartu University Hospital, Puusepa Str. 1A, Tartu, Estonia

## Abstract

**Background:**

Vitamin D has a wide variety of physiological functions in the human body. There is increasing evidence that low serum levels of this vitamin have an important role in the pathogenesis of different skeletal and extra-skeletal diseases. Vitamin D deficiency and insufficiency is common at northern latitudes. There are few population-based studies in the northern European region looking at the issue in a wider age group. We aimed to measure Vitamin D level in the general population of Estonia (latitude 59°N), a North-European country where dairy products are not fortified with vitamin D.

**Methods:**

The study subjects were a population-based random selection of 367 individuals (200 women and 167 men, mean age 48.9 ± 12.2 years, range 25–70 years) from the registers of general health care providers. 25-(OH) vitamin D (25(OH)D) level and parathyroid hormone (PTH) were measured in summer and in winter. Additionally age, sex, body mass index (BMI) and self-reported sunbathing habits were recorded.

**Results:**

The mean serum 25(OH)D concentration in winter was 43.7 ± 15 nmol/L and in summer 59.3 ± 18 nmol/L (p < 0.0001). In winter 73% of the subjects had 25(OH)D insufficiency (25(OH)D concentration below 50 nmol/L) and 8% had deficiency (25(OH)D below 25 nmol/L). The corresponding percentages in summer were 29% for insufficiency and less than 1% for deficiency. PTH reached a plateau at around 80 nmol/L. BMI and age were inversely associated with 25(OH)D, but lost significance when adjusted for sunbathing habits. A difference in the seasonal 25(OH)D amplitude between genders (p = 0.01) was revealed.

**Conclusion:**

Vitamin D insufficiency is highly prevalent throughout the year in a population without vitamin D dairy fortification living at the latitude of 59°N.

## Background

Vitamin D plays an important role in calcium and bone metabolism. Low levels of vitamin D lead to compensatory elevation of parathyroid hormone (PTH), which can cause lowering of bone mineral density (BMD) and eventually osteoporosis with fragility fractures or osteomalacia. Epidemiological studies have demonstrated a predisposition of the individuals with low levels of vitamin D to certain extra-skeletal diseases [1-4].

Vitamin D inadequacy is being increasingly recognised worldwide [5,6]. This shortcoming in vitamin D status is most prevalent in the elderly population, but affects people of all age groups [7-9]. Vitamin D serum concentrations are influenced by several modifiable and non-modifiable factors such as diet, latitude, season, time outdoors, skin pigmentation, clothing and tanning habits [10,11]. At high northern latitudes (above 40°N) even with adequate sun exposure the dermal generation of vitamin D is low or missing in winter and thus increases demand for dietary intake [5,12]. As very few foods naturally contain vitamin D in amounts satisfying such increased demand in winter, this results in marked seasonal variation in the levels of vitamin D [13]. It has been hypothesised that annually recurring cycles of vitamin D can cause mild secondary hyperparathyroidism, which leads to variations in bone turnover rate [14]. Such fluctuations could influence the rate of age-related bone loss [15] and might hamper peak bone mass attainment [16].

Different cut-off values for the normal threshold for vitamin D have been used until recently. A level of 50 nmol/L has been widely used to define 25(OH)D insufficiency, while some studies have used 37.5 nmol/L as the lowest level of sufficiency [17-19]. Recent studies, however, suggest that a 25-(OH)D level as high as 75 nmol/L or higher is needed to cover all physiological functions of vitamin D and should therefore be considered optimal [7,20-23].

There are wide regional differences in the 25(OH)D levels with an observed latitude effect, which has already been addressed in several countries by implementing dairy fortifications policies. Therefore, Norway and Sweden, two of the northernmost countries in Europe, have population 25(OH)D serum levels equal to or higher than those in Spain and Turkey, where sun is plentiful [7,24,25]. In Finland low 25(OH)D concentrations were reported in winter, although vitamin D dietary intake recommendations were met in the studied population [26]. Since then, dairy fortification has been implemented also in Finland. There are few population-based studies looking at seasonal differences of vitamin D concentrations and no data is available on vitamin D status of the Estonian population, which is one of the northernmost non-fortifying European countries [7,14,18,26-33]. The aim of the current study was to investigate vitamin D status and its seasonal dynamics in a random selection of the adult Estonian population.

## Methods

### Subjects

The study was conducted in Väike-Maarja municipality in Estonia in 2006. Study subjects were randomly selected from the registers of general practitioners in the region. An initial invitation and a second invitation (if needed) were sent to a total of 402 subjects to participate in the study. Of those invited, 243 (60%) responded. The non-responders were substituted once with the next person of the same age and sex from the patient register, in order to retain the population structure of the first selection. A total of 158 substitutions were made and an invitation (and a repeat if needed) was sent to them. An additional 124 subjects responded (response rate 79%) and were included in the study. A total of 367 subjects (200 women and 167 men, aged 25–70 years) participated in the study with a response rate of 66%. No inclusion/exclusion criteria were applied. An informed consent was obtained from all the subjects who participated. The Ethics Committee of Tartu University approved the study. All the 367 responders were subsequently invited to a follow-up blood test in the end of summer. Of the 367 subjects 316 (86%) complied and a second blood sample was taken in September. Self-reported summertime sunbathing habits and for women menopausal status were recorded with a self-administered questionnaire. The former was categorised as: usually bathes the whole body; bathes only the arms and face or avoids bathing totally. In addition, the use of vitamin D supplements was recorded. Subject weight and height were measured for body mass index (BMI; kg/m^2^) calculation.

### Laboratory measurements

Fasting overnight blood samples were obtained twice during the year, in winter (sampling time from January to March) and in the end of summer (September). All the samples were taken after overnight fasting between 8 AM and noon using pre-cooled tubes. Serum was separated and the samples stored at -20°C until analysed. The serum 25(OH)D level was measured in duplicates by radioimmunosorbent assay by DiaSorin (former Incstar), Stillwater, Minnesota, USA. The intra- and interassay CVs were 4.1% and 5.7%, respectively. For group discrimination, we used 25 nmol/L as the critical value for deficiency and 50 nmol/L as the cut-off value for insufficiency. 75 nmol/L was considered to be the optimal 25(OH)D level. Serum PTH was measured with an Immulite 2000 analyser (DPC). The intra- and interassay CVs were 10.5% and 8.2%, respectively.

### Statistics

The variables were verified for normality (Shapiro-Wilk test) and skewed variables (vitamin D in summer, PTH in winter and in summer) transformed using a natural logarithm for analysis. However geometric means are provided for better interpretation in the text. Student t or Mann-Whitney tests or analysis of variance (ANOVA) with Tukey test procedure were used to compare means. Correlations between variables were tested using the Pearson product-moment correlation test and presented as Pearson correlation coefficient (r_p_). A 5% probability for type I statistical error was allowed in all analyses (p < 0.05). The relationships between serum 25(OH)D concentration and PTH in summer were studied with the nonlinear least-squares regression method for optimal vitamin D cut-off determination. The locally weighted polynomial regression modelling (LOWESS) was used to model the winter PTH and vitamin D data. Determinants of 25(OH)D were studied using the multiple linear regression method with backward selection using 25(OH)D (models for summer and winter separately) as the dependent variable. The initial variables included in the regression analysis were age, gender, BMI, fresh milk consumption (as dL/day), other milk product consumption, caffeinated and alcoholic beverage consumption, smoking (both as a factor and number of cigarettes/day), vitamin D supplement usage, sun-bathing habits and menopausal status (in women). All variables with p < 0.1 were included in the final models. Statistical analysis was performed using the R software package version 2.7.0 (R: A language and environment for statistical computing. R Foundation for Statistical Computing, Vienna, Austria).

## Results

The mean age of the study group was 48.9 ± 12.2 years (range 25–70 years). All participants were mobile, community dwelling Estonians and Caucasians by race. The study group characteristics are outlined in Table 1.

**Table 1:** Characteristics and vitamin D status indicators of the study group (mean +/- SD).

	Females (n = 200)		Males (n = 167)		
	
	Mean	SD	Mean	SD	p value
Age	49.2	12	48.5	11.6	NS
BMI	28.5	6.9	27.9	4.6	NS
25(OH)D winter (nmol/L)	44.6	15.8	42.7	14.0	NS
PTH winter (pmol/L)	4.3	1.8	3.8	1.9	p < 0.05
25(OH)D summer (nmol/L)*	58.4	17.7	60.5	18.5	NS
PTH summer (pmol/L)*	3.9	1.7	3.6	1.7	NS

* n = 316; 138 males, 178 females

The mean 25(OH)D concentration in winter was 43.7 ± 15.0 nmol/L (17.2 ± 5.9 μg/L). A significant increase was observable in 25(OH)D concentration in summer to 59.3 ± 18.0 nmol/L (23.3 ± 7.1 μg/L) (p < 0.0001). The average increase of 25(OH)D for men was 17.9 nmol/L (relative increase 40%) and for women 13.4 nmol/L (30%), resulting in a difference in seasonal increase of 4.5 nmol/L (p = 0.01, 95% CI 0.6–8.2). Gender difference in the seasonal parathyroid hormone change was not significant. Eight subjects (2%), all women, were regularly using vitamin D supplements (over the counter vitamin D or cod-liver oil; 200–800 IU of vitamin D/day). Those subjects taking supplements had a higher serum concentration of vitamin D in winter (53.9 nmol/L in supplementers vs. 43.5 nmol/L in non-supplementers, p = 0.053).

**Figure 1:**
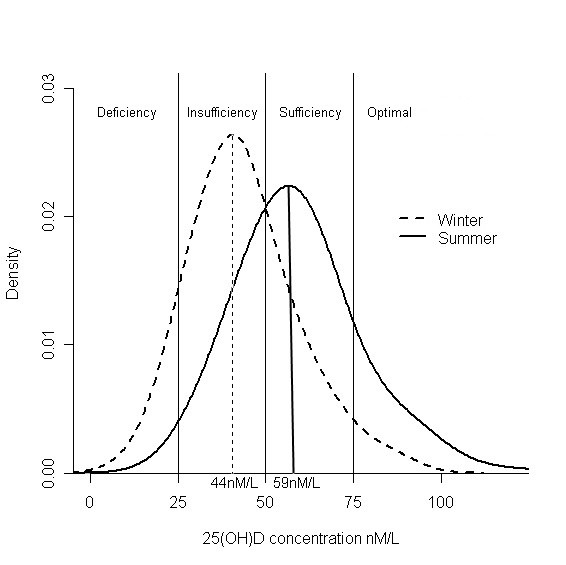
**The distribution of serum 25(OH)D in winter and in summer**.

Eight percent (n = 29) of subjects had vitamin D deficiency in winter, with equal gender distribution (15 women and 14 men). In summer less than 1% (n = 2; a single woman and a man) were still vitamin D deficient. In winter 73% of subjects (133 men and 135 women) had 25(OH)D level under 50 nmol/L, indicating insufficiency. In summer 29% (n = 93) of subjects were vitamin D insufficient. In winter 97% and in summer 87% of the subjects were below the optimal vitamin D level of 75 nmol/L (Figure 1). The mean 25(OH)D in winter for subjects refusing the follow-up in summer was lower (39.9 nmol/L) than that for subjects with both samples performed (44.4 nmol/L, p < 0.05).

**Figure 2:**
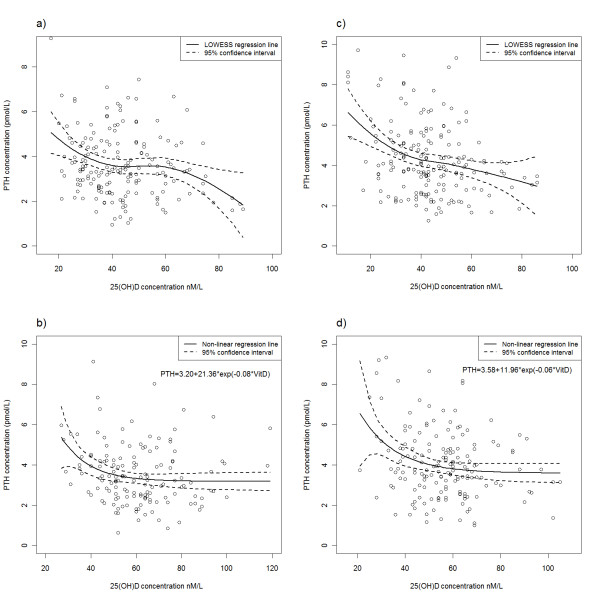
**25(OH)D plotted against serum PTH concentration**: a) winter, age under 50 years; b) summer, age under 50 years, c) winter, age 50 and above; d) summer, age 50 and above.

Serum PTH had a strong negative correlation with 25(OH)D concentrations both in winter and in summer (r_p _= -0.18 and r_p _= -0.19 respectively, p < 0.001). In summer the nonlinear least-squares regression shows an approximate cut-off value (PTH reaches a plateau) for 25(OH)D around 80 nmol/L in both younger (aged under 50 years) and older individuals (50 years and above). In winter there is no plateau effect in either of the age groups and PTH persists to decline even around the highest vitamin D values measured in the period (Figure 2). Hyperparathyroidism was present in 23 patients in winter (15 women and 8 men, mean 25(OH)D 35.9 nmol/L) and in 15 subjects in summer (8 women and 7 men, mean 25(OH)D 42.8 nmol/L). In winter 74% (17 of 23) and in summer 80% (12 of 15) were aged 50 or over. In winter 78% (18 out of 23) of the subjects with hyperparathyroidism were vitamin D insufficient and 26% (6/23) were vitamin D deficient. In summer 50% of these subjects were vitamin D insufficient, but none showed serum levels of deficiency. Three of the subjects with hyperparathyroidism had the condition persistent throughout the year.

There was a difference in the 25(OH)D level in summer between pre- and post-menopausal women (55 nmol/L vs. 61.5 nmol/L; p = 0.01) which was missing in winter. Age also correlated negatively with 25(OH)D only in the summer (p = 0.016). Both these associations lost significance in regression analysis if adjusted for self-reported sunbathing habits. The 25(OH)D level was strongly dependent on sunbathing habits both in summer and in winter (Table 2).

**Table 2:** Mean 25(OH)D according to sunbathing habits.

	25(OH)D in summer (nmol/L)			25(OH)D in winter (nmol/L)		
	
	N*	Mean	SD	N*	Mean	SD
Avoids sun	36	44.7	1.9	40	33.5	1.9
Arms/face	66	54.7	2.0	78	40.6	1.5
Whole body	206	63.1	1.3	240	46.3	1.0
P value (ANOVA)	P < 0.0001			P < 0.0001		

Arms/face vs. avoids sun^†^	10.1 (1.8–18.3); p = 0.01			7.0 (0.5–13.6); p = 0.03		
Whole body vs. avoids sun^†^	18.4 (11.2–25.6); p < 0.001			12.8 (7.1–18.6); p < 0.001		
Arms/face vs. whole body^†^	8.4 (2.7–14.0); p = 0.001			5.8 (1.4–10.1); p = 0.006		

* 10 subjects did not provide their sun-bathing habits data

† Post-hoc analysis using Tukey test; presented as differences in mean (95% CI); p value

The BMI and 25(OH)D correlated inversely: subjects with high BMI (>30) had lower 25(OH)D than subjects with a BMI under 30 (41.0 nmol/L vs. 44.8 nmol/L; p < 0.05). If a BMI cut-off point of 34 was used the difference was even more obvious (38.8 nmol/L vs. 44.5 nmol/L; p < 0.01). In multiple-regression analysis when adjusting for sunbathing habits BMI lost significance in winter. In analysis of variance (ANOVA) body mass index was significantly higher in the subjects who avoided sunbathing, and those who bathed the arms and face only when compared with total body exposers (p < 0.0001). Younger individuals were considerably more likely to sunbathe the whole body than older individuals (p < 0.0001).

Sunbathing habits (p < 0.0001), smoking (p = 0.008) and vitamin D supplement usage (p = 0.07) were the significant determinants of winter vitamin D level in multiple regression analysis explaining 12% of its variance (r^2 ^= 0.12). In summer the determinants for vitamin D levels were sunbathing (p < 0.0001), smoking (p = 0.001) and BMI (p = 0.03) explaining 18% of its total variance (r^2 ^= 0.18).

## Discussion

The study is one of the few population-based studies on vitamin D status in northern Europe. The mean 25(OH)D in winter is well below 50 nmol/L, with only a third of the Estonian population showing sufficient vitamin D levels and just 3% of the population above the optimal level. Although the mean 25(OH)D level was around 60 nmol/L in summer, with 2/3 of the population reaching sufficiency, the majority of the subjects (around 90%) still stayed below the optimal level of 75 nmol/L. The finding is similar to the results of other studies in the region demonstrating that the summer build-up of 25(OH)D is inadequate in northern countries [26].

For overview purposes the currently available vitamin D status data in similar populations performed to date is given in Table 3. Due to the inter-assay and also inter-laboratory variability of different vitamin D measurement techniques some caution is advocated in interpretation [34]. At 44 nmol/L the winter mean 25(OH)D concentration in Estonia is comparable with levels in several southerly situated countries but falls short if compared with vitamin D levels in Belgium, France, Switzerland and the US [7,7,14,18,26,29-33] (Table 3). Our summer 25(OH)D is the lowest in this comparison, which corresponds well with the latitude effect. The fact that there is a low proportion of vitamin D supplement users in Estonia and the diet here is also scarce in natural sources of vitamin D (such as fish or fish products) [35] makes the population largely dependent on the vitamin D stores accumulated during the sunny season. In a country populating an even higher latitude, Norway, a study showed that 40% of the population regularly uses cod-liver supplements, almost 60% consumes fish liver products and 57% other vitamin D supplements, beside cod liver oil [36]. This might explain why Norway, despite being located at 65–71 N is one of the countries with the highest vitamin D levels in Europe.

**Table 3:** 25(OH)D status in population studies of adults (15+) in the order of latitude.

Location	Latitude	Method	N	Population representation (subjects per capita)	Vitamin D (nmol/L), mean and/or range		Deficiency % <25 nmol/L		Insufficiency % <50 nmol/L	
					
					summer	winter	summer	winter	summer	winter
New Zealand [30]	35–47S	RIA Diasorin	2946	1:1,400	67	44	0–10		48	
Japan [31]	35 N	RIA Disaorin	197	1:647,000	79	38	ND	ND	1	87
US [32]	25–47 N	RIA Disaorin	18462*	1:16,200	62–90	60–79	1–3	1–5	8–34	13–40
Italy [14]	40 N	RIA Disaorin	90	1:645,000	84	43	2 **	18 **	4	70
Switzerland [33]	46–47 N	CPB Amersham	3276	1:2,300	ND	50 †	ND	6 †	ND	34–95 †
France [7]	43–51 N	RIA Incstar	1569	1:39,000	ND	61 (43–94)	ND	14 **	ND	75 ***
Germany [29]	49 N	RIA Incstar	415	1:199,000	67–70	40–45	~5 ††	30–40	ND	ND
Belgium [18]	50 N	ChL Diasorin	126	1:82,000	ND	48	ND	ND	ND	34 †††
**Estonia**	**59 N**	**RIA Disaorin**	**367**	**1:3,600**	**59**	**44**	**<1**	**8**	**29**	**73**
Finland [26]	60 N	RIA Incstar	328	1:16,000	ND	46	ND	26–28	ND	56–86 ***

* Two different seasonal sub-populations; ** level of <30 nmol/L used to define deficiency; *** own specific cut-off for insufficiency (based on PTH curve) was used; † data from October to June (no summer months); †† no official data in paper (calculated from a figure); ††† level of <37.5 nmol/L was defined as insufficient; ND – no data.

The proportion of 25(OH)D deficiency and insufficiency in our study population is comparable with other countries (table 3) during different seasons even though it is geographically the highest northern country in the comparison after Finland. However we consider the percentages too high to be satisfactory, as there is still a large proportion of the population who especially during winter are at an increased health risk. Addressing these shortcomings is important as the health risks include diseases like osteoporosis, rickets and with some evidence supporting benefits or risk reductions also for several types of common cancers, diabetes, multiple sclerosis and atherosclerosis [1-4].

Our study supports the evidence that PTH achieves a plateau at 25(OH)D concentrations between 75 and 90 nmol/L demonstrated recently [21]. In winter no plateau was achieved, since the majority of observations (97%) were below 75 nmol/L.

Our study demonstrated an unexpected finding that men have a higher amplitude of 25(OH)D variance through the seasons than women. Clothing habits have been shown to influence 25(OH)D levels [11,37]. There might be gender-specific clothing differences (although not of a culture-religious nature in the studied region) which are responsible for a higher 25(OH)D in men after the sunny season. There is also a possibility that men are more likely to work outside during the summer than women, resulting in longer exposure to UV light. Secondly, fat tissue is the physiological depot for vitamin D suggesting that the obese have an increased storage capacity of 25(OH)D [38,39]. The fact that with equal BMI and age, men have a lower body fat percentage than women [40] might explain the faster decline and also the faster accrual in men. Studies have also shown that vitamin D has a role in neuromuscular function [41,42]. This might mean that men, having higher muscle mass and lower fat tissue [40], require more vitamin D to sustain muscle cell function and in the absence of UVB radiation, deplete their vitamin D stores faster.

The study showed that BMI, age and menopause determined 25(OH)D status in the end of summer, but not in winter. The fact that all these associations lost significance when adjusted for sunbathing habits suggests that most probably it is not the weight and age *per se *influencing 25(OH)D, but their effect on sunbathing, which impacts the UVB doses they are subjected to in summer, resulting in lower 25(OH)D levels. This is plausible, as we found no correlation between 25(OH)D and BMI, age or menopausal status (an indirect indicator of age for women) in winter, when these factors cannot exert their effect on sun exposure.

As a study limiting factor we might be reporting 25(OH)D values, which are slightly higher than the average year to year measurements at this latitude, as in summer 2006 the sun was comparatively plentiful compared to previous years. In our study people with a higher 25(OH)D value in winter were more likely to attend the follow-up test in summer, which is another minor aspect of bias. Therefore it is possible that we might be overestimating vitamin D levels in summer. However, since the drop-out rate was not remarkable, this bias should not influence the results significantly. At the time of the study our laboratory was not part of the international vitamin D assays quality assessment scheme (DEQAS) which, as already mentioned, would have enhanced the comparability of the results with those performed by other laboratories and assay methods.

## Conclusion

We demonstrated that 25(OH)D levels are low all year round in Estonia, a country situated at a high northern latitude. Dairy fortification with vitamin D (which is already practiced in neighbouring Finland, Norway, Sweden and also the US) might address these shortcomings in addition to propagating healthy sunbathing habits, since sun had a major effect on vitamin D status and explained much of the age and BMI caused variance in 25(OH)D levels.

## Competing interests

Authors Mart Kull, Riina Kallikorm, Anu Tamm and Margus Lember have no conflicts of interest. This work was possible thanks to the support of the Estonian Science Foundation (Grant 6452) and an unrestricted grant from Merck Sharp and Dohme used exclusively to cover laboratory analysis costs.

## Authors' contributions

MK is the drafter of the current paper. He was actively involved in the designing and coordination of the study, material acquisition, analysis and interpretation of the material. RK and ML were actively involved in the designing of the study, material collection, and interpretation of results, drafting and revising the manuscript as well as acquisition of the funding for the study. In addition ML was the general supervisor of the research group. AT was involved and responsible for laboratory handling and analysis of blood samples, participated in the critical review of the paper. All the authors named have read and approved the final version of the manuscript before submitting.

## Pre-publication history

The pre-publication history for this paper can be accessed here:


